# Linking Small-Scale Flight Manoeuvers and Density Profiles to the Vertical Movement of Insects in the Nocturnal Stable Boundary Layer

**DOI:** 10.1038/s41598-020-57779-0

**Published:** 2020-01-23

**Authors:** Charlotte E. Wainwright, Don R. Reynolds, Andy M. Reynolds

**Affiliations:** 1 Rothamsted Research, Harpenden, Hertfordshire, AL5 2JQ UK; 2 Natural Resources Institute, University of Greenwich, Chatham, Kent, ME4 4TB UK; 3 Now at Department of Civil and Environmental Engineering and Earth Sciences, University of Notre Dame, Notre Dame, IN 46556 USA

**Keywords:** Agroecology, Animal migration

## Abstract

Huge numbers of insects migrate over considerable distances in the stably-stratified night-time atmosphere with great consequences for ecological processes, biodiversity, ecosystem services and pest management. We used a combination of meteorological radar and lidar instrumentation at a site in Oklahoma, USA, to take a new look at the general assistance migrants receive from both vertical and horizontal airstreams during their long-distance flights. Movement in the nocturnal boundary layer (NBL) presents very different challenges for migrants compared to those prevailing in the daytime convective boundary layer, but we found that Lagrangian stochastic modelling is effective at predicting flight manoeuvers in both cases. A key feature for insect transport in the NBL is the frequent formation of a thin layer of fast-moving air – the low-level jet. Modelling suggests that insects can react rapidly to counteract vertical air movements and this mechanism explains how migrants are retained in the jet for long periods (e.g. overnight, and perhaps for several hours early in the morning). This results in movements over much longer distances than are likely in convective conditions, and is particularly significant for the reintroduction of pests to northern regions where they are seasonally absent due to low winter temperatures.

## Introduction

Migration is a key life-history component in many insects with important ecological and evolutionary consequences for the species, as well as significant economic, environmental and cultural impacts on humankind (e.g. refs. ^1–6^). Insect migration can take a number of forms^4^, but movement over any significant distance is usually wind-aided following ascent high into the air^7^. Migratory flights at altitudes above the insect flight boundary layer(i.e. the iso-velocity surface ~1–10 m above the ground at which the wind speed is equal to the insect’s airspeed^8^) will be strongly influenced by the state of the atmospheric boundary layer (ABL) into which the migrants launch themselves; this will particularly apply to small insects with their very low airspeeds. The ABL is the layer of the atmosphere that is directly affected by the Earth’s surface, and it is approximately 1 km deep during the daytime and 100–200 m deep at night (see ref. ^9^ for a detailed description). In the daytime, migrants will usually enter the convective boundary layer (CBL), the portion of the ABL where the vertical air motion is dominated by thermally-driven updrafts and downdrafts, and the (quite subtle) behavioral responses of small insects to vertical air movements under these conditions was the subject of a previous paper (see ref. ^10^). There we reported that insects are moving downwards through the downdrafts and are moving upwards when in the updrafts albeit at a slower pace than the air itself.

Migrants that continue to fly, or that takeoff, at dusk will usually find themselves in very different conditions – those of the nocturnal stable boundary layer (NBL) where the flow is much less turbulent than during the day. On clear evenings, radiative cooling of the surface cools the air above it so that temperatures tend to *increase* with height (i.e. an inversion forms) and the statically stably-stratified temperature regime tends to suppress updrafts and downdrafts^9^. Above the NBL is a nearly neutrally stable residual layer, the remnant part of the previous daytime’s CBL; nocturnal insect migration also takes place here. Migrating insects can use the stable stratified atmosphere to make undisturbed long-range downwind migrations which may persist for long periods during the night, often in layers of strong wind which can transport them rapidly over considerable distances (several hundreds of km per night)^2,7,11^.

If air temperatures are reasonably conducive to insect flight (above, say, ~10°C; see 11 and references therein), a mass take-off and ascent around dusk is virtually ubiquitous, and has been recorded by all radar systems capable of detecting insect targets (see 7, Chap. 10 and 15 in^11,12^). The general view is that emigrants ascending at this time will get no help from updrafts and must therefore climb to high altitude by sustained active flight^12,13^. In addition, particularly in warmer areas of world, some small, typically day-flying, migrants (such as aphids) may continue flying after dark^7,14–17^. They then have to maintain themselves in flight, sometimes for hours, by their own efforts, notwithstanding the factthat they are strongly dependent on convective updrafts to assist in their ascent when engaged in (more typical) daytime migration^10^.

Though the nocturnal stable boundary layer does not have strong up- and downdrafts there still exist regions of sinking and rising air, which at longer timescales can be caused by large-scale convergence and divergence. At shorter timescales wave-like atmospheric structures are often seen within the NBL, including gravity waves, vorticity waves, etc., as well as so-called ‘dirty’ waves that are only approximately periodic and may have varying amplitude and wave period (see ref. ^18^ for a comprehensive review on wave-turbulence interactions relevant to the NBL). Other vertical motions in the NBL may result from the combination of the shutdown of turbulent mixing at sunset occurring over a laterally-varying buoyancy field, which can produce weak but persistent ascent of magnitude 3–10 cm s^−1^ alongside a strong nocturnal Blackadar-type low-level jet horizontal wind speed profile^19^.

Although the amplitude of vertical air motion in the NBL is significantly reduced as compared to the daytime convective boundary layer, there exists a need to determine the effect of these motions on nocturnal insect migration and, more generally, to compare the behavioral responses of small insects to vertical air movements throughout the diel cycle of the ABL. Knowledge of how insects react to different vertical air movements is a necessary step in understanding their altitudinal selection and improving insect movement forecasting models.To realize this objective, we used a combination of zenith-pointing Doppler lidar and Ka-band dual-polarized profiling cloud radar which together provide precise measurements of the vertical component of air velocity concurrently with a quantification of the movements of insects aloft at various times of diurnal cycle^10^.

Here we investigate the general behavioural responses of insects to air movements under stable NBL conditions by measuring the velocities of more than 2.95 million insect targets, relative to the vertical motion of the air in which they are flying, from a site in Oklahoma, USA. This Great Plains location is situated in the ‘Mississippi flyway’ where nocturnally-migrating insects ride warm southerly nocturnal low-level jet winds, easily covering distances of several hundred kilometres in a night’s flight^15,20^. This phenomenon is of considerable agricultural importance because it facilitates the annual invasion, every spring and summer, of the northern Great Plains states of USA and Canada by economically significant pests (leafhoppers, aphids and moths) which cannot overwinter in this region^15,20–22^. Low-level jets are also important for long-distance spread of insect pests in other parts of the world^23,24^.

Previously, we have found that Lagrangian stochastic modelling is an effective way to account for small insect movements in convective boundary layers^10^. Here we show that this modelling approach can also account for insect movements in stable boundary layers. We show that our theory can symmetrically and mechanistically link together characteristic features of the insect flight behaviours (responses) to known flow features in the stable boundary layer as well as the convective boundary layer.

## Method and Observational Results

The data used in this study encompasses 1^st^ July–31^st^ August 2015. This 2-month interval was selected to minimize additional radar clutter from migrating birds, and is the same period as that investigated by Wainwright *et al*.^10^. Here we are concerned with insect flight in the nocturnal stable boundary layer rather than the daytime convective boundary layer as examined previously.

The methods used herein largely follow those used in ref. ^10^, which were based on a modified version of the analysis used by Geerts and Miao^25,26^. The vertical air motion was provided by a zenith-pointing Halo Streamline pulsed Doppler lidar (Halo Photonics, Malvern, UK) located at the Atmospheric Radiation Measurement program Southern Great Plains (SGP) site in Lamont, Oklahoma, USA. The SGP site is located at 36.605°N, 97.485°W and is at an altitude of 318 m above mean sea level. The topography is flat and the habitat is dominated by rangeland. During July and August 2015 when this study takes place, the average daily high temperature was 32.3 °C and the average night-time low was 20.4 °C. The data provided by the Doppler lidar does not contain returns from insect motion and provides the true vertical motion, *w*_*a*_, of the background flow in which insects in the boundary layer are embedded at temporal and spatial resolutions of 1.2 s and 26 m, respectively. A co-located Ka-band (8.6-mm wavelength) zenith-pointing cloud radar (ProSensing Inc., Amherst, MA, USA) also measures vertical motion, here denoted *w*_*r*_, but this contains the motion of the insects superimposed on the background flow. The *w*_*r*_ data from the Ka-band radar has temporal and spatial resolution of 2.7 s and 30 m respectively. The spatial and temporal resolution of the remote sensing instruments are considerably higher than any other existing instrumentation which can sense insect motion over a period of several weeks or months. By comparing the vertical motion with and without insect ‘contamination’ we are able to derive the component due to the motion of the insects alone, *w*_*i*_, from simple subtraction via *w*_*i*_ = *w*_*r*_ − *w*_*a*_. Throughout this paper we will use the convention of positive values of *w* representing rising air or insect motion and negative values representing subsidence or descent.

**Figure 1:**
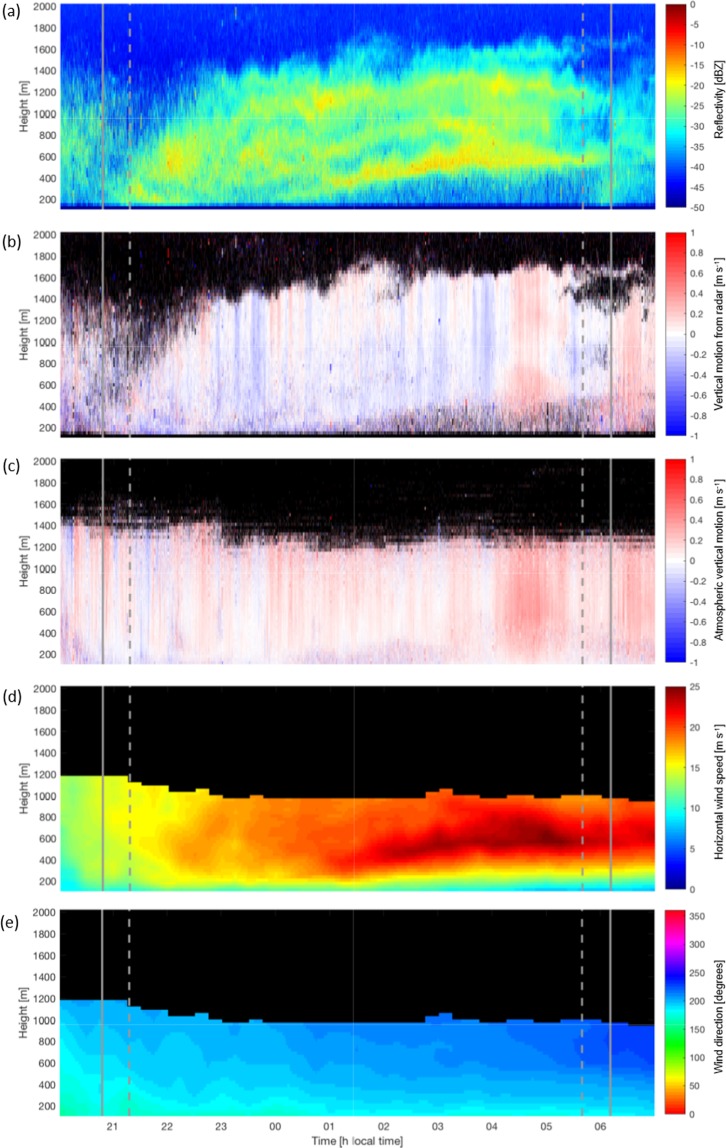
An example case from10–11 July 2015. (**a**) Time-height plot of reflectivity [in dBZ] measured by the Ka-band radar. (**b**) Vertical motion, *w*_*r*_ [in m s^−1^], recorded by the radar. Panel (c) shows the atmospheric vertical motion, *w*_*a*_ [in m s^−1^], recorded by the collocated Doppler lidar. Panel (d) shows the horizontal wind speed [in m s^−1^] and (**e**) shows the wind direction [in degrees] derived from the Doppler lidar data. The solid grey lines indicate the time of sunset and sunrise and the dashed lines represent the onset and cessation of civil twilight.

In addition to providing vertical motion, the Ka-band radar also measures vertical profiles of reflectivity, *Z*. In cloud- and precipitation-free air the reflectivity can be used as a proxy for animal density in the airspace. Comparing reflectivity at different altitudes and across different nights allows us to see when the migration intensity is heaviest and at what heights migrating insects are flying. Time-height profiles of *Z*, *w*_*r*_, and *w*_*a*_ can be seen for one example case of 10–11 July 2015 in Fig. 1a–c.

Horizontal wind speed and direction are also of interest in potentially influencing insect vertical movement. These were calculated from the Doppler lidar, which performs a plan position indicator scan once every fifteen minutes using eight equally spaced azimuth angles aligned to the cardinal directions. A velocity azimuth display (VAD; see ref. ^27^) technique is then applied to derive vertical profiles of the horizontal wind speed and direction. In Fig. 1d,e the wind speed and direction have been interpolated in time and height to match the resolution of the cloud radar data.

### Average nocturnal insect vertical motion

In addition to investigating the response of insects to the surrounding airflow, we also examined how the average vertical motion of insects varies with time and altitude over the course of the night.

**Figure 2:**
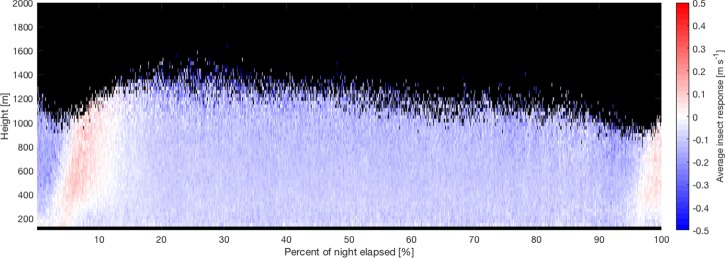
Time-height plot of the *w*_*i*_ values (representing the insects’ unaided vertical movements) averaged across the 62-day observation period. The *x*-axis shows the percentage of the night elapsed, with 0% representing sunset and 100% representing sunrise. The time is split into quantiles to account for differing day length across the study period. Blue represents insect descent and red represents ascent. There is clear evidence of mass ascent shortly following sunset and again following sunrise.

Prior to this analysis the Doppler lidar data was interpolated in time and height to match the resolution of the Ka-band radar data. Periods of precipitation were removed using a linear depolarization ratio (LDR) threshold following Martner and Moran^28^ with a threshold value of −15 dB. Meteorological scatterers have low LDRs while biological scatterers have much higher values. The LDR data was examined on a day with both insect movement and precipitation (see ref. ^10^) and we found that the insect data had LDR values between −10 and −21.4 dB (5^th^ and 95^th^ percentile) while the corresponding LDR range for precipitation was −21 to −22.7 dB. Here, we select the −15 dB threshold to ensure that no precipitation is included in the analysis, although some insect data may also be removed in this filtering procedure. Data showing no evidence of insect contamination were removed using a co-polar signal-to-noise ratio (SNR) filter of 0 dB, as in Wainwright *et al*.^10^. In order to account for the changing day length over the two-month study period, each night was split into 1000 quantiles between sunset and sunrise. Insect response values falling within each quantile and 30-m height bin were calculated by subtracting *w*_*a*_ from *w*_*r*_ as described above, and the resulting values of *w*_*i*_ were then averaged for each bin. The resulting nightly time-height profiles of *w*_*i*_ were combined by taking the median value across the 62 days. The resulting average time-height profile of *w*_*i*_ across the study period is shown in Fig. 2. Since the lidar height coverage is variable depending on atmospheric conditions, only quantiles with data for at least 30 of the 62 nights are shown in the figure. In Fig. 2 and throughout the remaining analysis data from the whole two-month study period are considered together without regard for possible variations in migration patterns over that time.

The average insect response in Fig. 2 shows slight overall descent throughout most of the night, and mean *w*_*i*_ between the 20^th^ and 90^th^ centiles of the night is −0.115 m s^−1^ with a standard deviation of 0.045 m s^−1^. There is also clear evidence of mass ascent shortly following sunset and again around sunrise. We also see slightly stronger descent directly preceding the two periods of ascent. In other words, there is the expected pattern of day-flying insects descending around sunset, followed by the mass take-off and ascent of nocturnal insects which then continue to migrate for varying periods through the night. Just before dawn,the nocturnal insects still in flight tend to descend and land and then there is a conspicuous take-off of dawn crepuscular flyers. (Note that this dawn activity is quite distinct from daytime flight associated with boundary layer convection which gradually develops from mid-morning onwards, as surface heating promotes convection^10^). The presence of the anticipated main daily features in insect flight activity provide a check on the integrity of the observational protocols.

**Figure 3:**
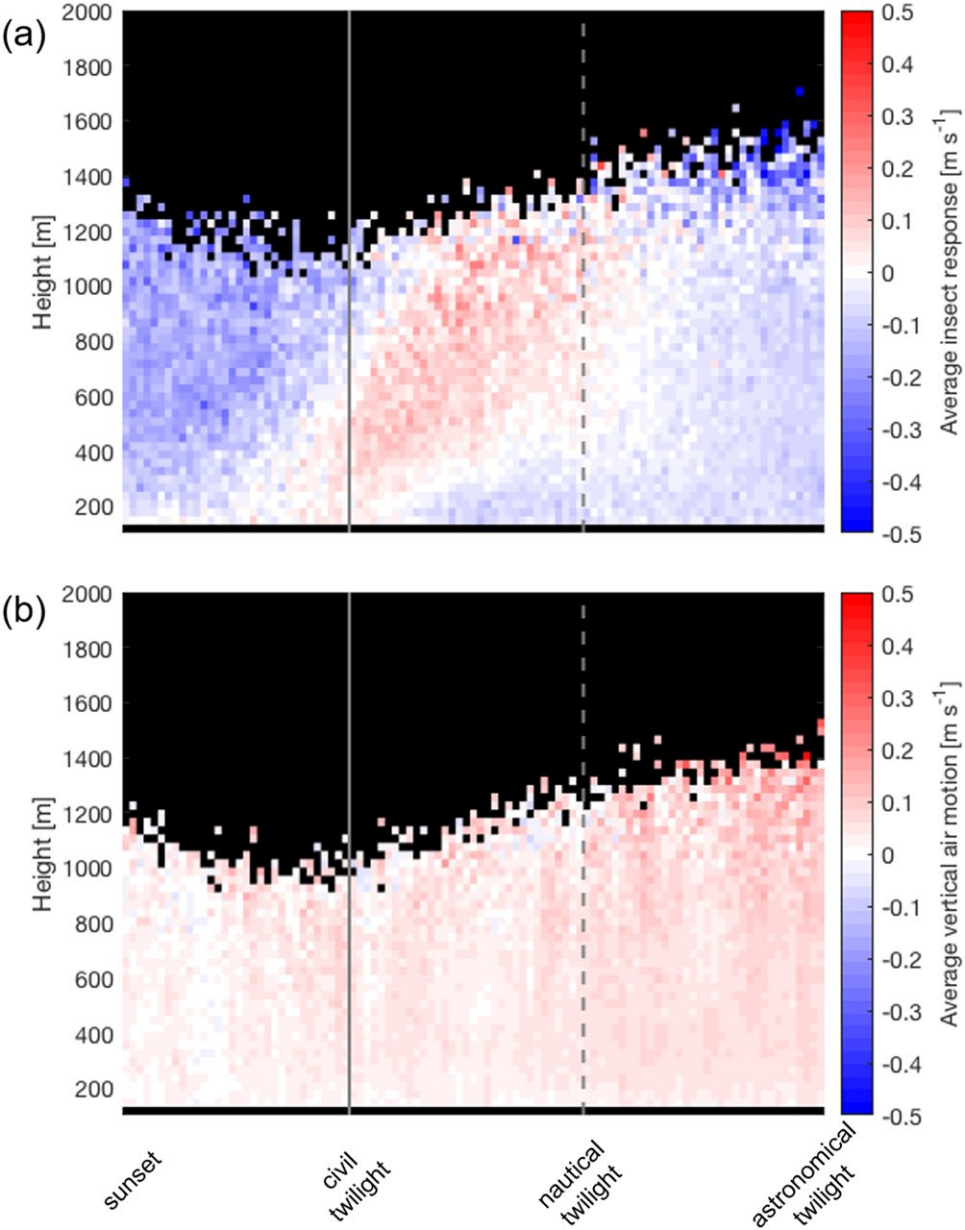
(**a**) Time-height plot of the average *w*_*i*_ values between sunset and astronomical twilight. The *x*-axis is divided into thirds by civil twilight (solid grey line) and nautical twilight (dashed grey line). (**b**) Same as (**a**) but for vertical air motion *w*_*a*_.

The dusk ascent of insects is further investigated in Fig. 3, which shows *w*_*i*_ (Fig. 3a) and *w*_*a*_ (Fig. 3b) for times between sunset and astronomical twilight, averaged across the two-month period. The time is evenly split into thirds marked by civil and nautical twilight to highlight the insect response with respect to decreasing daylight. The initial ascent from low levels is seen to begin very shortly after sunset (and we note that no data is available at heights below 100 m). The ascent continues at increasing elevations from civil twilight until nautical twilight. The median *w*_*a*_ value between sunset and civil twilight is 2.1 cm s^−1^, between civil and nautical twilight it is 3.5 cm s^−1^ and between nautical and astronomical twilight it is 5.7 cm s^−1^. The corresponding value for the 30 minutes before sunset (not shown) is 0.8 cm s^−1^. We also calculate an average *w*_*i*_ value representing the three periods shown in Fig. 3 by taking the median *w*_*i*_value between 600 and 800 m heights for the middle 50% of each time period. These height and time intervals were selected to capture the main ascent between civil twilight and nautical twilight. The resulting median *w*_*i*_ values were −15.4 cm s^−1^ between sunset and civil twilight, 7.3 cm s^−1^ between civil and nautical twilight, and −5.7 cm s^−1^ between nautical and astronomical twilight.

### Response of small insects to surrounding airflow

The main focus of our investigation is how small insects respond to the surrounding vertical motion in the stable boundary layer. In the previous section, data from the whole night covering sunset to sunrise was presented, butwe now restrict our analysis to 23:00–04:00 local time (04:00–09:00 UTC). This time period is at least two hours after sunset (latest sunset during the study was 20:53 local time/01:53 UTC) and two hours before sunrise (earliest sunrise 06:15 local time/11:15 UTC), so should encompass only the stable NBL without residual effects from the evening or morning transition regimes. The corresponding time period used for the fully-developed and well-mixed CBL in ref. ^10^ was 14:00–18:00 local time (19:00–23:00 UTC).

Since here we are interested in elucidating the responses of individual insects, further filtering beyond that described in the previous section is necessary to remove instances of multiple insects in the beam. This is accomplished using a spectrum width filter of 0.1 m s^−2^ in addition to the LDR and SNR threshold filters described above. Further details on the filtering can be found in ref. ^10^.

**Figure 4:**
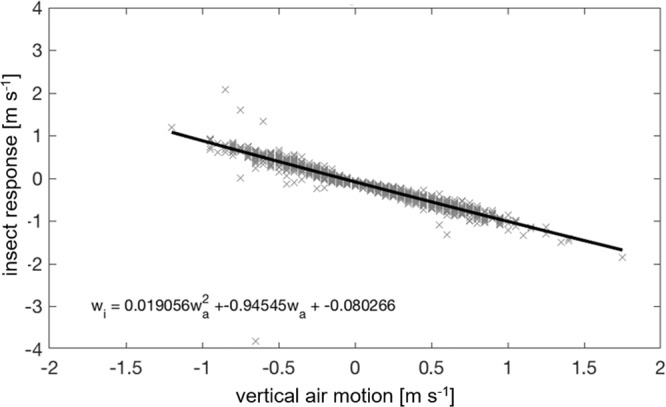
The derived insect response in vertical motion to the vertical motion of the surrounding airstream for the nocturnal boundary layer between 23:00–04:00 local time on 10–11 July 2015 over Lamont, Oklahoma, USA. The solid black line represents the best fit to the data, performed using quadratic linear regression. The linear (negative) relationship suggests that the insects are opposing any upward and downward air motions almost exactly, thus ensuring they stay at their preferred altitude (for example, in the layers seen in Fig. 1).

For ease of comparison with ref. ^10^, we follow the technique used by Geerts and Miao^25^ and split the insect response, *w*_*i*_, into bins based on the surrounding air motion *w*_*a*_. The data are examined at 6-minute intervals as in refs. ^10,25^. This 6-minute duration was originally selected for considerations regarding the turnover time of eddies in the convective boundary layer and is kept here for consistency. The data in each 6-minute time bin is separated into *w*_*a*_ bins of size 0.05 m s^−1^ with maximum and minimum values of ±2 m s^−1^. All *w*_*i*_ measurements falling within each *w*_*a*_ bin are then averaged to give a single *w*_*i*_ value for each velocity and time interval. An example case for 10–11 July 2015, corresponding to the period 23:00–04:00 illustrated in Fig. 1 is shown in Fig. 4.

The example case for 10–11 July 2015 in Fig. 1 shows distinct layering of insects in the airspace, which are clearly visible in the reflectivity (Fig. 1a). The formation of multiple layers of insects in the nocturnal stable boundary layer is well documented (e.g., refs. ^29,30^), and cases with up to five distinct layers have been recorded^31,32^. The occurrence of multiple insect layers is more common in warmer regions where the flight ceilings may be at much higher altitudes^11^. For the case shown in Fig. 3, the 10 °C isotherm was not reached until a height of 3.2 km, and so any effective flight ceiling would be above the data considered herein.

The wind speed and direction (Fig. 1d,e) indicate the presence of a strong southerly low-level jet (LLJ) above the southern Great Plains, with supergeostrophic wind speeds of up to 25 m s^−1^ around 600 m height. Southerly LLJs occur frequently in this region and are particularly common during spring and summer^33–35^, and the frequent presence of southerly LLJs are exploited by aerial migrants on their journeys northwards from overwintering grounds to summer breeding areas^15,20,36–38^. From around 02:00 onwards, the lowest and densest layer of insects visible in Fig. 1a is seen to coincide well with the highest wind speeds, i.e., the region of the jet nose (Fig. 1d).

The method described in the previous section was used to examine the insect response to changing vertical motion in the 10–11 July 2015 case shown in Fig. 1. The resulting relationship between *w*_i_ and *w*_*a*_ is illustrated in Fig. 4. For this case there is an almost inverse relationship of the insect response to the vertical air motion, indicating that the response of the insects is to oppose any vertical motion at such a rate to negate any changes in altitude. This is also reflected in the relative constancy of the insect layers with height seen in Fig. 1a. Further evidence for this comes from the average insect response for the whole two-month period; during the time after nautical twilight (i.e. once the main dusk ascent is over) this was found to be −5.7 cm s^−1^, exactly balancing the median *w*_*a*_ value of 5.7 cm s^−1^ for this period.

### The continuation of daytime (convective boundary layer) migration into the night

**Figure 5:**
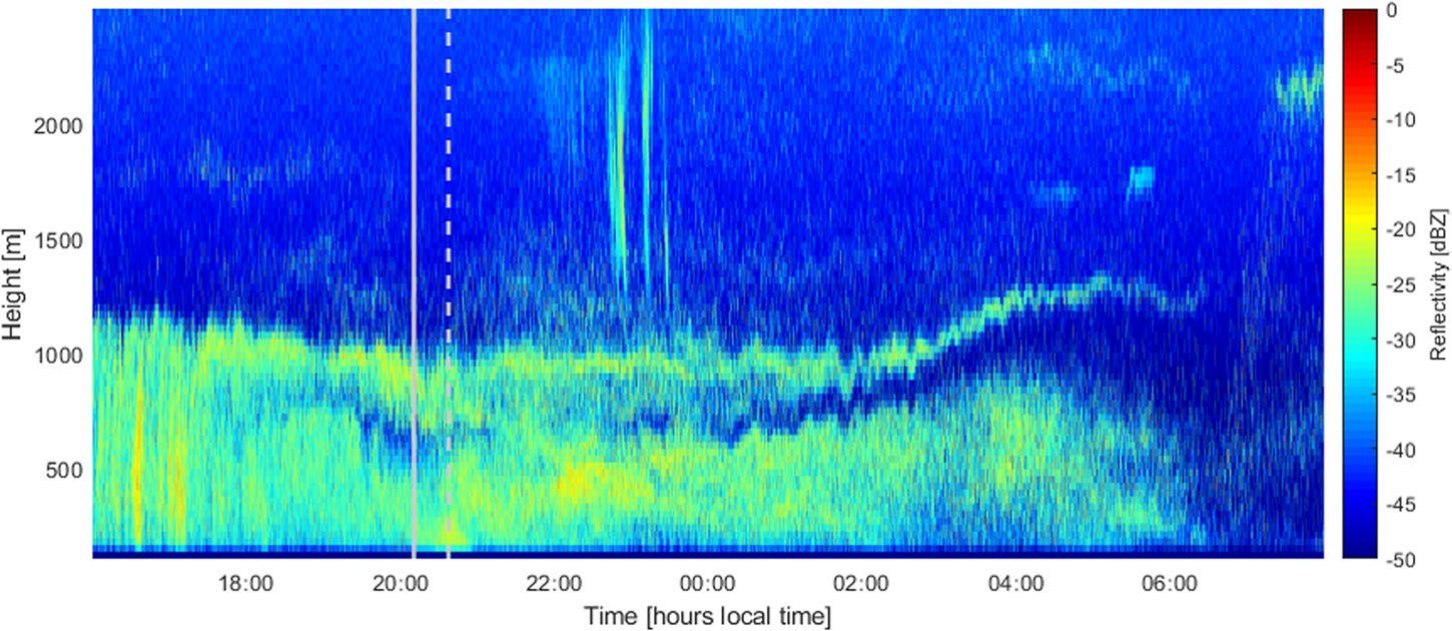
Time-height plot of reflectivity [in dBZ] recorded by the radar on the night of 23–24 August 2015. The solid grey line indicates local sunset and the dashed line the onset civil twilight.

As mentioned above, the long-range pest invasions of the northern Great Plains region from south-central USA (over distances of up to ~1000 km, and flight durations of 12 hours or more), are greatly facilitated if day-flying migrants transit across the dusk period into the NBL. Although they are usually weaker than the layers that form later on in the night, we sometimes see layers of small insects persisting after sunset. There are also cases with strong layers persisting right across the twilight period, typically in cases of fairly heavy migration with high reflectivity. An example of such a case is shown in Fig. 5, which shows the reflectivity on the night of 23–24 August 2015. Although there is an indication of additional insect ascent between sunset (solid grey line) and civil twilight (grey dashed line), a strong insect layer at around 1000 m persists from several hours before sunset, across sunset, and through the night. The temperature at this height was 18 °C at 19:00 local time and remained above 16 °C at 07:00 the following morning.

## Data Access

The Ka-band radar and Doppler lidar datasets analyzed in the present study are available in the DOE ARM Climate Research Facility repository at https://www.arm.gov/capabilities/instruments.

The Oklahoma Mesonet data references in the Discussion is available via DOI 10.15763/dbs.mesonet.

The radiosonde data was accessed via the University of Wyoming Upper Air website at http://weather.uwyo.edu/upperair/sounding.html.

## Theory

**Figure 6:**
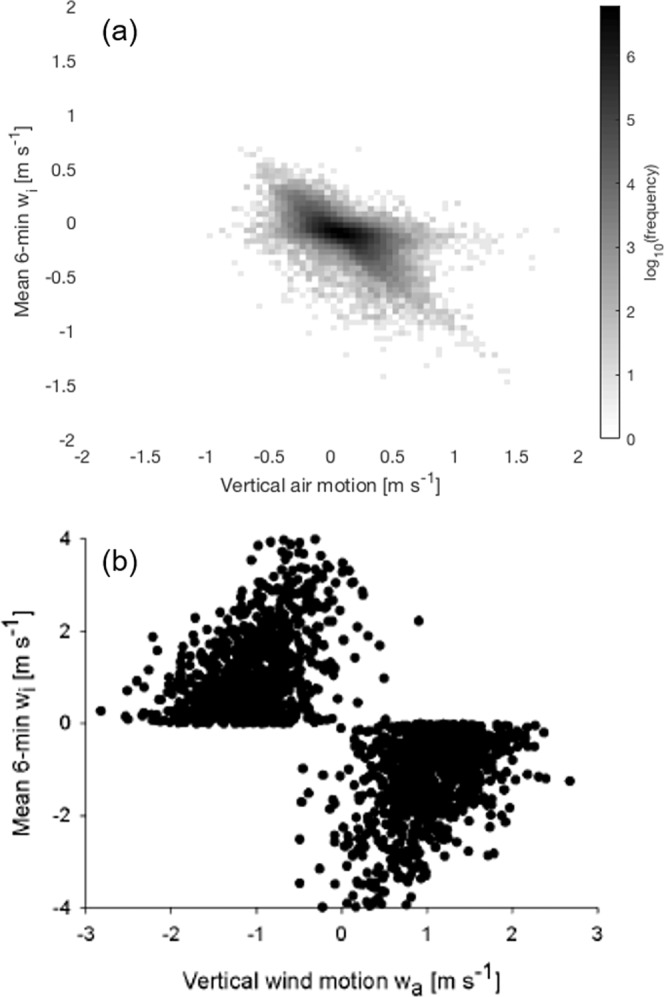
(**a**) A heatmap of the derived insect response in vertical motion to the vertical motion of the surrounding airstream for the nocturnal boundary layer. The heatmap shows the frequency of occurrence for the insect response in log scale. (**b**) An example of a simulated insect response. The scatter is the result of randomly sampling positions from a Gaussian aerial density and from a bi-Gaussian distribution of velocities, Eq. 1. Predictions are shown for the case when updrafts and downdrafts are present in equal numbers (*A* = 1/2)).

We previously showed how the insect flight response (i.e., the difference between the insect’s vertical velocity and that of the surrounding air currents) can be deduced mathematically from insect aerial density profiles and the velocity statistics of the vertical air movements^10^. We showed that the typical response in a convective boundary-layer is well represented by a simple quadratic function of air velocities (and, in fact, some further findings related to *fully convective boundary-layers* can be seen in Supplementary Material 2). This prediction applies irrespective of atmospheric stability and so is consistent with our new observations for stable boundary-layers (Fig. 4). Our modelling approach can, however, also be used to make more nuanced predictions that can serve as more stringent tests of the model. Here we use the modelling approach to predict complex responses resulting from the presence of updrafts and downdrafts, coherent flow features that are known to be present sporadically in nocturnal boundary layers^39^. We thereby show how insect responses (Fig. 6a) can be directly and simply linked to physical characteristics of the turbulent flows they are flying through. To do this we assume that vertical air movements due to the presence of regions of upward and downward air motion can be characterized by bi-Gaussian velocity distributions,

1 $$P\left({w}_{a}\right)=\frac{1}{\sqrt{2\pi }\sigma }\left[A\exp \left(-\frac{{\left({w}_{a}-{\overline{w}}_{u}\right)}^{2}}{2{\sigma }^{2}}\right)+\left(1-A\right)\exp \left(-\frac{{\left({w}_{a}-{\overline{w}}_{d}\right)}^{2}}{2{\sigma }^{2}}\right)\right],$$

where *A* and 1-*A* are the relative proportions of upward and downward motion, ${\overline{w}}_{u}$ and ${\overline{w}}_{d}=-\,A{\overline{w}}_{u}/(1-A)$ are their average velocities and $\sigma$ is their root-mean-square velocity. Following Luhar and Britter^40^ such distributions have been used widely and successfully when predicting turbulent dispersal in convective boundary layers. Here, however, we are concerned with making qualitative rather than quantitative comparisons with our observations. For such bi-Gaussian velocity distributions, our theory^10^ predicts that the accelerations of small insects and the surrounding air differ by an amount

2 $$\overline{A}\left({w}_{a},z\right)=\frac{1}{\rho }\frac{d\rho }{dz}\left[A\frac{{\overline{w}}_{u}}{2}\left(erf\left(\frac{{w}_{a}-{\overline{w}}_{u}}{\sqrt{2}\sigma }\right)+1\right)/P\left({w}_{a}\right)+\left(1,-,A\right)\frac{{\overline{w}}_{d}}{2}\left(erf\left(\frac{{w}_{a}-{\overline{w}}_{d}}{\sqrt{2}\sigma }\right)+1\right)/P\left({w}_{a}\right)-{\sigma }^{2}\right]$$

where $\rho$ is the aerial density profile of insects that characterises how the average concentrations of insects varies with height, *z*. This additional acceleration represents a driving force towards higher aerial densities that allows for the maintenance of non-uniform aerial density profiles. Without this force, gradients in aerial densities would eventually get smoothed out as there would be nothing to counter the tendency of turbulent dispersal to drive small insects upwards (i.e., towards lower aerial densities). The acceleration term, Eq. 2, can be regarded as encapsulating an active response of small insects to the surrounding air flow causing an additional change in velocity, ${w}_{i}=\overline{A}({w}_{a},z)dt$, beyond that caused by following the air flow, where *dt* is the time over which accelerations remain significantly correlated. When we go beyond ref. ^10^ and make the additional assumption that insects tend to be concentrated in the upper half of the layer when updrafts (or lower half when downdrafts) are present, model predictions (Fig. 5b) are broadly consistent with our observations. These findings show that our theory can attribute characteristic features of the insect flight behaviours (responses) to known flow features.

## Two Case Studies

As further tests of our model we applied to it two nights, averaging over the period from midnight to 03:00. On both occasions, 11 July and 18 July, updrafts predominated over downdrafts, occurring around 80% of the time (see Supplementary Figs. A,B). These weak but persistent nocturnal ascents might be caused by the same circumstances that result in the frequent low-level jet over Oklahoma^19^. Application of our methodology^10^ to the test cases is straightforward, but because of the weak ascent, results in a complicated set of governing equations which appear to be analytically intractable. [The equations are greatly simplified when, as the case of a daytime convective boundary^10^, the mean velocity of the vertical air motions is zero]. Here the governing equations were solved for the insect response, *w*_*i*_, as a function of the velocities of the vertical air motions, *w*_*a*_ (Supplementary Figs. C and D). Flow conditions are encoded in the first four moments (equivalently the mean, variance, skewness and flatness) of the distribution of vertical air movements. These are used as model inputs. Convective flows with strong updrafts and downdrafts have a strongly skewed distribution of vertical motion. Stable flows have *w*_*a*_ distributions that are nearly Gaussian. Model predictions compare favourably, capturing accurately differences in the responses on the two nights. The response was convex on 11 July and concave on 18 July when the amplitude of the vertical air motions was greater. The form of the predicted response is sensitively dependent on the skewness and flatness of the vertical air motions.

## Discussion

Our general objective in these studies has been to investigate the precise behavioural responses of small migrant insects to the motion of the air in which they are flying, under two very different atmospheric regimes,the day-time convective boundary layer (in the previous paper^10^), the night-time SBL (in the present work).

One of the main features of the present observations (Fig. 2) is the significant upward motion seen shortly following sunset, representing mass take-off of insects at dusk. This behavior, stimulated by changes in illumination level, is almost universally recorded by insect-detecting radars^11^ as long as the temperature threshold for migratory flight is exceeded for sufficient taxa of migrant insects. Small insects are not necessarily dominant in this dusk emigration. Our Ka-band radar returns mean that we can detect insects down to about aphid-size (~0.5 mg), but there is no way to automatically distinguish small and large insects in our data – a small insect at the centre of the radar beam will give a similar return as a large insect away from the beam centre. Nonetheless, the fact that dusk ascent is well underway by 20 min after sunset suggests that small insects are certainly there in numbers, because the larger insects tend to take off a little later when it is becoming dark^7,11^.

The average vertical air motion during this time is close to zero (upwards at ~0.03 m s^−1^ in the hour after sunset) which is about a tenth of the unaided ascent rates (~0.2 m s^−1^) of which small migrant insects are capable^41^ and our data shows a median insect ascent rate of 0.07 m s^−1^ during the main period of dusk ascent (Fig. 3) across the full two-month period investigated. This validates previous assumptions that small insects emigrating at dusk actively climb to altitude with minimal atmospheric assistance^13^, in stark contrast to small insect migration in the well-mixed daytime convective boundary layer which relies on assistance from thermals^10^. Figure 2 also shows a second period of insect ascent at dawn, although this is less strong than the clear ascent signal seen at dusk. As mentioned previously, this is a true dawn ascent (probably triggered by changes in illumination) rather than insects taken up in convective updrafts. Significant dawn ascents are recorded relatively infrequently in temperate regions as they are limited by the threshold temperature for insect take-off. We examined temperature data from the nearby Oklahoma Mesonet station in Medford, OK^42,43^ and found that the average daily minimum temperature over the two-month period was 20.7°C, which is well above the threshold required for insect take-off. Similar dawn ascents have also been recorded in several previous studies conducted in the tropics and sub-tropics^13,30,44^, and weaker dawn ascents have also been recorded in northern Europe^45^ during the summertime when temperatures are sufficiently high.

Insect layers formed in the stratified early-morning atmosphere (arising from dawn emigration or even from all-night flight^15^) sometimes persist for several hours but are usually disrupted by the upward progression of convective turbulence^45–48^. This strong layering effect in the SBL has previously been suggested to correspond to insect layers forming at heights of localized temperature or wind speed maxima^10^. Discerning whether temperature or wind is the primary driver of insect layer formation has been the subject of previous studies, with mixed results^25,34,49^, and is complicated by the fact that wind and temperature maxima are often collocated so disentangling the role of each variable is not always possible. In this study the formation of insect layers was observed to frequently correspond with the presence of the low-level jet, with the densest layers of insects often collocated with the highest wind speeds in the jet maximum (as in Fig. 1a,d). Inspection of the dataset reveals that this is generally the case, at least earlier in the season. Later in the season the situation becomes more complex as the southerly LLJ acts to hinder any southward ‘return’ migration. Further discussion of this situation is outside the scope of the present paper, but we note there is often significant directional wind shear between the LLJ and the surrounding air, and insect behavior seems to vary depending upon the wind speed and direction within, above, and below the LLJ. The placement of insect migrants within a nocturnal jet nose region has also been demonstrated by Wolf *et al*.^49^ and Beerwinkle *et al*.^50^, and it has been suggested that the formation of insect layers at wind speed maxima may be caused by a turbophoretic effect due to the relative reduction in wind shear associated with the wind speed maxima^51^. The exact wind speed and direction conditions at the heights of the higher layers of insects are unknown as the lidar data does not reach this altitude, but we see that the density of insects is increased throughout the entire depth of the low-level jet compared to the regions above and below. The higher layers of insects may have different preferred flight temperatures, may be comprised of different species, or may have ascended from different localities.

Both the dawn and dusk mass ascents show a consistent signal at heights of up to at least 1 km. This is indicative of a lack of flight ceiling within the atmospheric boundary layer due to the high summertime temperatures in the observational region. This is further evidenced by the insect layer at around 1200 m shown in Fig. 1a, which persists throughout the night. The altitudes at which the insect layers form and the corresponding horizontal wind speed at these altitudes will have a major impact on the distance insects are able to travel over the course of a single migratory flight. Our observations also revealed examples where daytime, convection-associated, migratory flight (typical of aphids) was apparently extended through dusk twilight and into the night. If, after a certain amount of daytime migration, small insects become entrained in layers in the NBL, very long-distance movements are possible. As already noted, these have immense practical consequences in determining the extent and timing ofthe annual reinvasions of the northern Great Plains by aphids and leafhoppers which are virus vectors or direct pests of crops (see refs. ^15,20,22^, and references therein).

During the main part of the night, the insect response is an average downward motion (with respect to the surrounding air) of 0.115 m s^−1^. This means that insects in the main ‘transmigration’ phase (after their initial ascent) tend to oppose vertical atmospheric motions, so as to maintain a constant altitude, reflecting their entrainment in one of the observed atmospheric layers (Fig. 1a). The layers may correspond to different temperatures, wind speeds, or wind directions, and so it is possible that such layering reflects the varying optimal flight conditions of different taxa.

The close correspondence between the predicted and derived responses suggests that the insects may remain in the layers by responding rapidly to turbulent features of the wind stream, rather than local temperature maxima which are another putative driver for the formation of highly-structured, nocturnal density profiles (see Chap. 10 in^11,47,52^). A response to a wind-related feature rather than temperature seems particularly likely where (as here) air temperatures just below the jet altitude are still well above insect flight thresholds. In any event, these cues must be very strong to retain small insects like aphids, which do not remain at altitude during the day without some convective support. In the convective case insects are generally moving upwards when in the updrafts albeit at a slower pace than the air itself, and moving downwards through the downdrafts^10^. In more weakly-turbulent stable nocturnal conditions the response can, as we have shown, negate any changes in altitude due to air movements.

Our predictions were made using modified Lagrangian stochastic models for the simulation of tracer-particle trajectories in atmospheric boundary-layers. The modifications allow for the establishment and maintenance of the observed insect density profiles which are thereby linked to predictions for small-scale flight manoeuvers. This contrasts with previous studies which deduced density profiles from modified Lagrangian stochastic models by presupposing that insects have so-called ‘turbophoretic’ responses which result in their concentrating preferentially in turbulence minima^51^. Turbophoresis is the tendency of particles suspended in turbulence to drift down gradients in turbulent kinetic energy.

Our analysis and that of Wainwright *et al*.^10^ suggests that airborne dispersal of weak fliers across widely-varying atmospheric conditions can be predicted reliably on the basis of high-resolution aerial density profiles. Such data should become increasingly available from combinations of special-purpose entomological radars and operational weather surveillance radars. Recent technological advances in specialized insect monitoring radar have enabled insects’ vertical velocity to be derived from a single instrument for the first time, holding great promise for furthering the study of vertical motion of insects^12^, although this is presently limited to larger insect targets. Data accumulated over a series of seasons will allow the characterization of particular migration systems^11,53^, i.e. estimation of the probabilities of various migration events, associated parameters such as intensity, direction, heights of flight, likely displacement distance, etc., and correlations with environmental conditions. Attention should be directed particularly to migrations over very long distances which might spread pests and diseases well beyond their normal ambit. The development of millimetric entomological radars could drive the development of an operational (near real-time) warning service for migratory invasions of small insect pests (c.f. ref. ^54^).

## Supplementary information

Supplementary File 1.
