# Supplementary material for: Linking Small-Scale Flight Manoeuvers and Density Profiles to the Vertical Movement of Insects in the Nocturnal Stable Boundary Layer
Source: Sci Rep. 2020 Jan 23;10:1019. doi: 10.1038/s41598-020-57779-0 (PMC6978332; doi:10.1038/s41598-020-57779-0)
Supplement: Supplementary file 1 [file 41598_2020_57779_MOESM1_ESM.pdf]

Linking small-scale flight manoeuvers and density profiles to the vertical movement of insects in the nocturnal stable boundary layer

Charlotte E. Wainwright, Don R. Reynolds and Andy M. Reynolds

Supplementary Material

Supplementary Figures

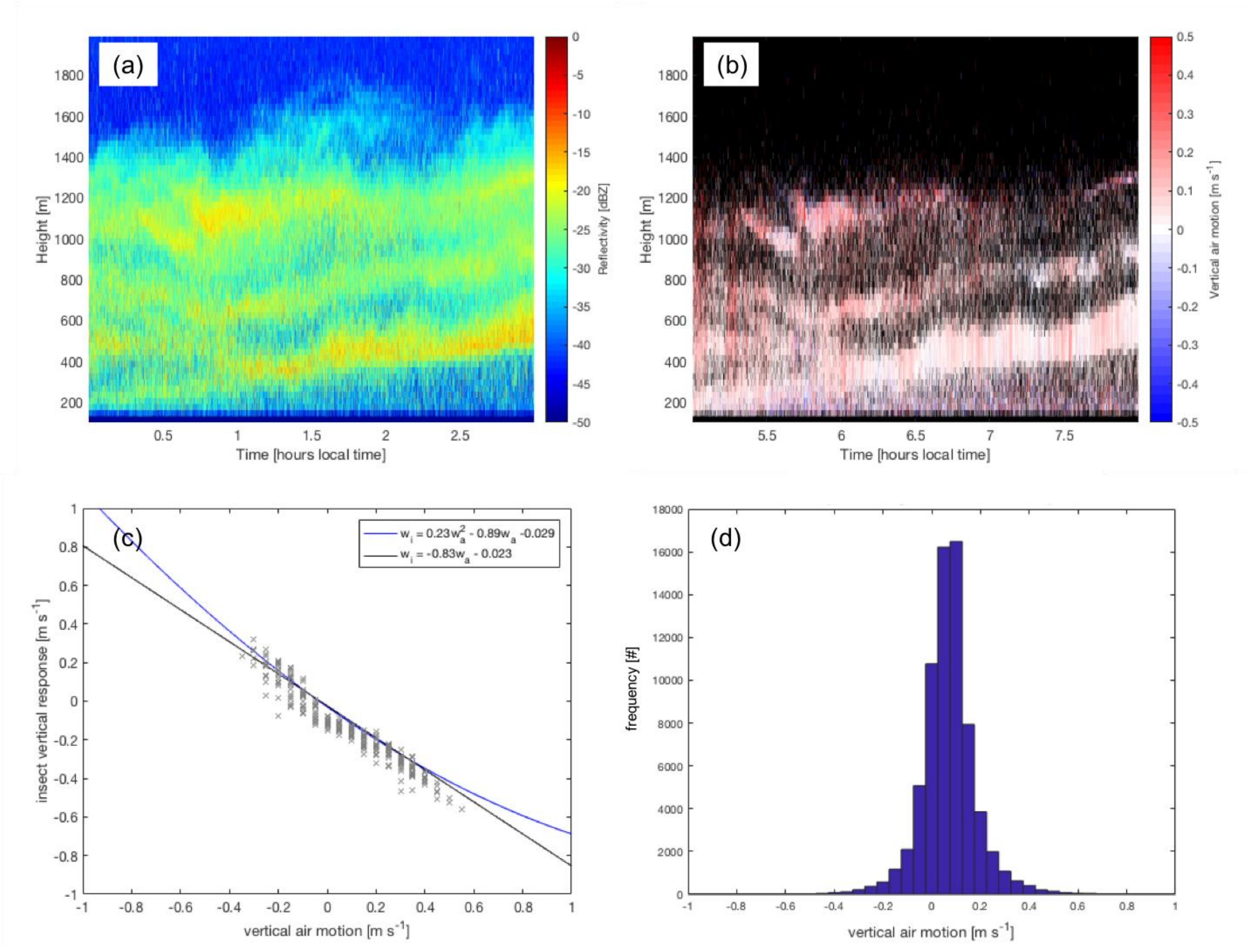

Supplementary Fig. A. Further details from an example case from July 11 2015. a) Time-height plot of reflectivity [in dBZ] measured by the Ka-band radar between 00:00 – 03:00 local time. b) Vertical motion, w_a [in m s^{-1}], recorded by the collocated Doppler lidar. c) Insect vertical response compared to the vertical motion of the surrounding air, with linear and quadratic best fit lines. d) Histogram of w_a recorded during the 3-hour period.

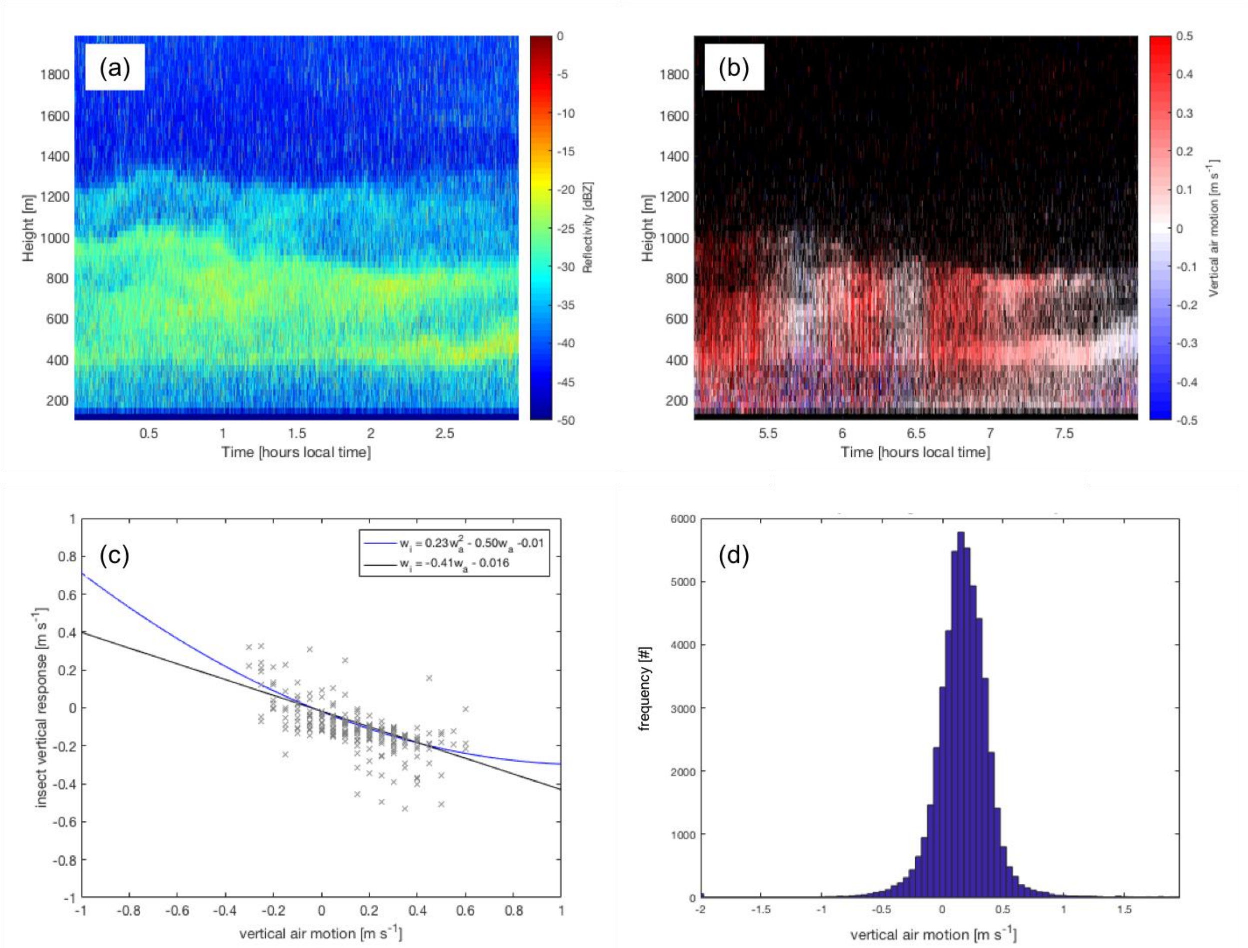

Supplementary Fig. B. As in Supplementary Fig. A but for 18 July 2015.

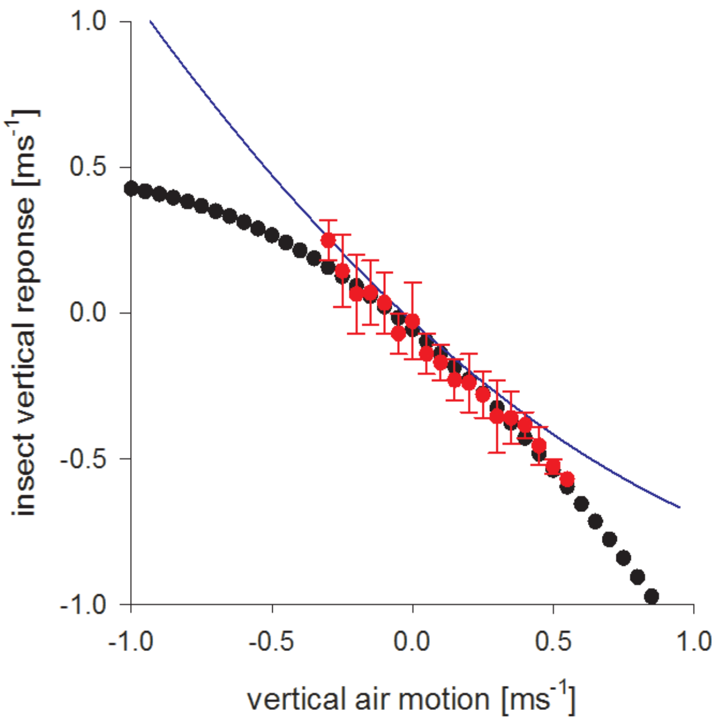

Supplementary Fig. C: The predicted insect response in vertical motion to the vertical motion of the surrounding airstream for the nocturnal boundary layer between 00:00 – 03:00 local time on 11 July 2015 over Lamont, Oklahoma, USA (•) together with the mean and range of the derived response (red symbols). The blue line shows the quadratic best fit from Supplementary Fig. A. panel (c). Predictions were obtained using the methodology given in ref. 9.

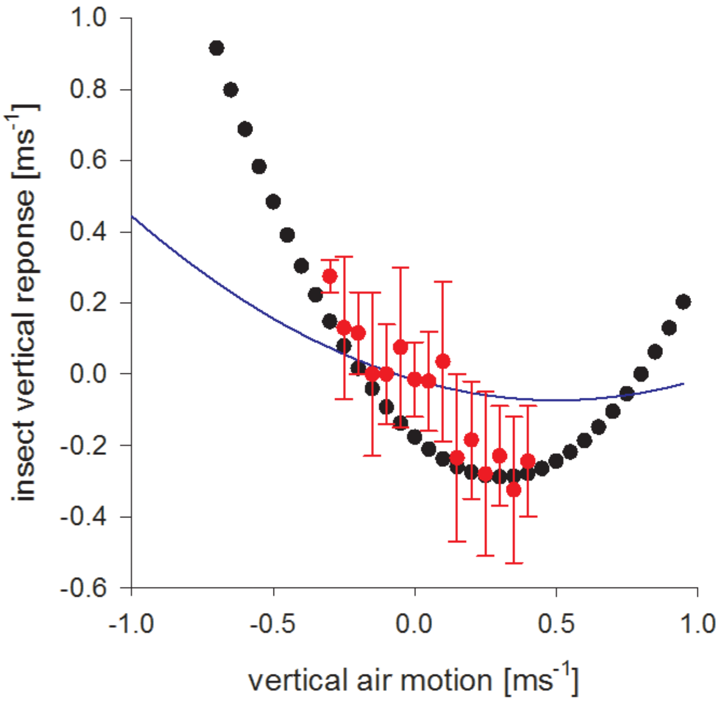

Supplementary Fig. D. The predicted insect response in vertical motion to the vertical motion of the surrounding airstream for the nocturnal boundary layer between 00:00 – 03:00 local time on 18 July 2015 over Lamont, Oklahoma, USA (•) together with the mean and range of the derived response (red symbols). The blue line shows the quadratic best fit from Supplementary Fig. B. panel (b). Predictions were obtained using the methodology given in ref. 9.

Supplementary Material 2: Further findings related to the *convective boundary-layer case.*

See Wainwright, C. E., Stepanian, P. M., Reynolds, D. R. & Reynolds, A. M. The movement of small insects in the convective boundary layer: linking patterns to processes. *Scientific. Reports.* **7**, 5438 (2017).

Height specific response functions for insects in fully convective boundary-layers.

In our previous paper (Wainwright *et al.*[9]) we presented predictions for the response function in the middle of a convective boundary-layer and we showed that these predictions are described by a simple quadratic (concave) function. Here consistent with a height-dependent analysis of our observations (Supplementary Fig. Y below) we report that the predicted response is height dependent, being concave in the lower half of the boundary-layer and being convex in the upper half where there are relative few insects (Supplementary Fig. Z).

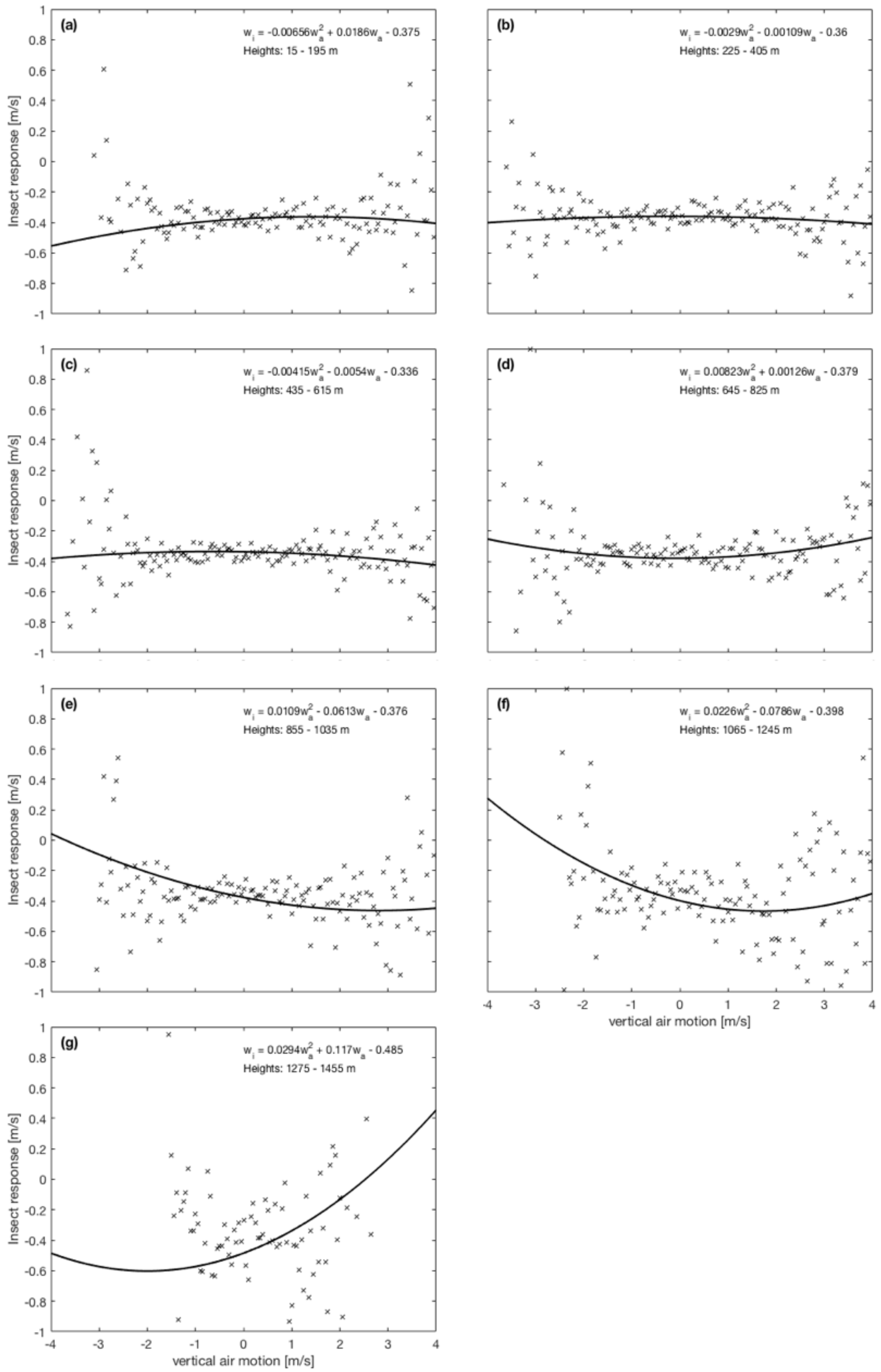

Supplementary Fig. Y. Observed difference between the vertical velocities of small insects and the surrounding airstream in the fully-developed convective boundary layer, based on 29,343 data points. The solid black lines indicate the quadratic best fits to the data. The fits were performed using a quadratic linear regression. Panels represent increasing data from increasing heights in 180-m increments.

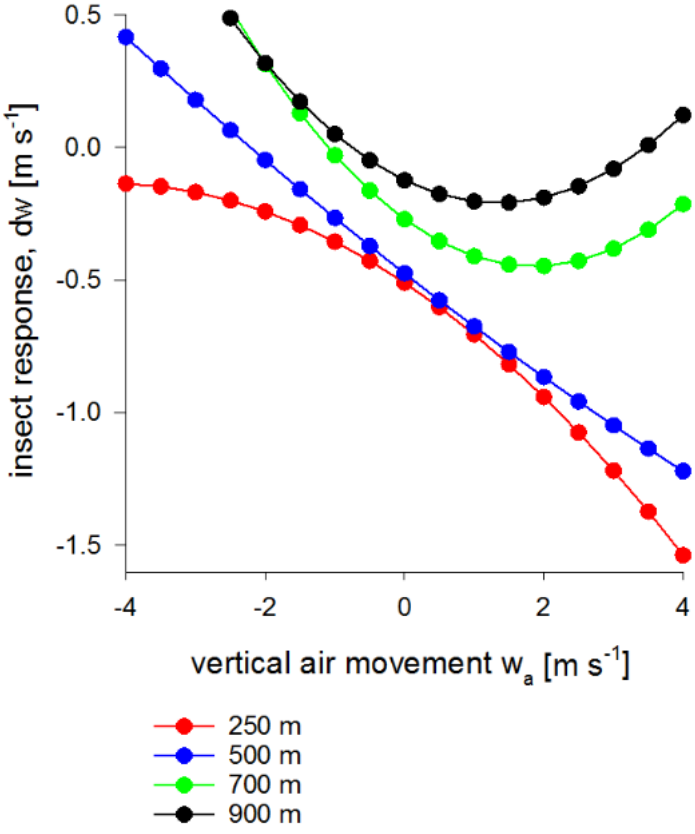

Supplementary Fig. Z. Predicted difference between the vertical velocities of aphid-size (~0.5 mg) insects and the surrounding airstream in a 1000-m high convective boundary layer.
